# 

**Published:** 1888-08

**Authors:** 


### The Chicago Medical Journal and Examiner.

Volume 57. Number 2.

### CONTENTS OF THIS NUMBER.

PAGE.

**Original Communications—**

Secondary Mixed Infection in Typhoid Fever. B. Holmes ........ 65
Four Cases of Surgical Kidney. I. N. Danforth ........ 74

**Editorial—**

Higher Medical Education ........ 83
New Antipyretics and Proprietary Medicines ........ 84
Contagious Affections ........ 85

**Society Reports—**

Transactions of the Gynæcological Society of Chicago ........ 86
Michigan State Board of Health ........ 99
The American Otological Society ........ 101
The American Ophthalmological Society ........ 103

**Foreign Medical Societies—**

Clinical Society of London ........ 104
Medico-Chirurgical Society of Edinburgh ........ 106
Berlin Medical Society ........ 113

**Foreign Correspondence** ........ 115
**Extracts and Abstracts** ........ 121
**Book Reviews and Notices** ........ 127
**List of Advertisers** ........ x
**Publisher's Announcements** ........ x

